# β-Hemoglobinopathies: The Test Bench for Genome Editing-Based Therapeutic Strategies

**DOI:** 10.3389/fgeed.2020.571239

**Published:** 2020-12-03

**Authors:** Gloria Barbarani, Agata Łabedz, Antonella Ellena Ronchi

**Affiliations:** Dipartimento di Biotecnologie e Bioscienze, Università di Milano-Bicocca, Milan, Italy

**Keywords:** β-hemoglobinopathies, genome editing, globin genes, hereditary persistence of fetal hemoglobin, programmable endonucleases

## Abstract

Hemoglobin is a tetrameric protein composed of two α and two β chains, each containing a heme group that reversibly binds oxygen. The composition of hemoglobin changes during development in order to fulfill the need of the growing organism, stably maintaining a balanced production of α-like and β-like chains in a 1:1 ratio. Adult hemoglobin (HbA) is composed of two α and two β subunits (α2β2 tetramer), whereas fetal hemoglobin (HbF) is composed of two γ and two α subunits (α2γ2 tetramer). Qualitative or quantitative defects in β-globin production cause two of the most common monogenic-inherited disorders: β-thalassemia and sickle cell disease. The high frequency of these diseases and the relative accessibility of hematopoietic stem cells make them an ideal candidate for therapeutic interventions based on genome editing. These strategies move in two directions: the correction of the disease-causing mutation and the reactivation of the expression of HbF in adult cells, in the attempt to recreate the effect of hereditary persistence of fetal hemoglobin (HPFH) natural mutations, which mitigate the severity of β-hemoglobinopathies. Both lines of research rely on the knowledge gained so far on the regulatory mechanisms controlling the differential expression of globin genes during development.

## Introduction

Historically, because of the abundance and accessibility of red blood cells, globins served as a model for major discoveries later extended to other genes. In 1967, hemoglobin was the first human complex protein crystallized (Muirhead et al., 1967); in 1980, the β-locus was the first cloned gene cluster (Fritsch et al., 1980) and soon became the prototypical model of tissue-specific and developmentally regulated genes. In 1987, the β-locus control region (LCR) was the first long-distance position-independent enhancer characterized (Grosveld et al., 1987), and the current looping model for the interaction of far apart regulatory regions owes much to the study of globin gene sequential activation during development (Stamatoyannopoulos, 1991; Fraser and Grosveld, 1998). The wealth of data accumulated on globin genes put them now at the frontline of development of genome-editing approaches with therapeutic purposes.

## The Globin Genes

In man, globin genes are organized in two clusters lying on chromosomes 16 (α cluster) and 11 (β cluster). A fine-tuned regulation maintains a 1:1 ratio of α-like and β-like chains during development to produce first HBZ (ζ2ε2, ζ2γ2), then HbE (α2ε2), HbF (α2γ2), and finally HbA (α2β2) together with a small amount of HbA2 (α2δ2), in a process called hemoglobin switching.

At the molecular level, the hemoglobin switching involves the establishment of sequential long-range chromatin physical interactions between a common LCR and the different globin promoters active at a given developmental time (with inactive genes being looped out) in a structure called active chromatin hub (ACH) (Carter et al., 2002; Tolhuis et al., 2002; Palstra et al., 2003). The formation of ACH requires the presence of transcription factors/cofactors that, by binding with the correct affinity to their consensus on DNA, creates the favorable condition for the expression of the gene of interest (Wilber et al., 2011).

## β-Hemoglobinopathies

Qualitative or quantitative defects in the β-globin production cause the most common monogenic diseases: sickle cell disease (SCD) and β-thalassemia (Weatherall, 2008; Thein, 2013); both diseases, in particular β-thalassemias, are very severe in homozygous subjects, whereas symptoms are mild in carriers. In SCD, the amino acid β6Glu>Val substitution leads to the formation of long hydrophobic polymers of HbS that precipitate within the cell under hypoxic conditions, conferring the typical sickle shape. Sickle cells tend to stick, causing vessel obstruction and, because of their fragility, they frequently undergo hemolysis, finally leading to anemia.

In β-thalassemias, a wide spectrum of mutations causes the reduction of β-globin, which can range in severity from total absence (β^0^) to partial reduction (β^+^). Causative mutations vary from large deletions to small insertions or deletions (indels) and point mutations within the β gene. β-thalassemia mutations impact on all the different steps of the β gene expression regulation (Thein, 2013): transcription (mutations within regulatory regions), RNA processing (splicing mutations), and translation (ATG mutations, non-sense and missense mutations). In rare cases, β-thalassemia is caused by mutations outside the β-locus, in genes involved in the basal transcription machinery XPD (Viprakasit et al., 2001) or in the erythroid-specific transcription factor GATA1 (Yu et al., 2002). The common output of β-thalassemia mutations is a reduced production of functional β chains with the consequent precipitation of the excess α chains causing hemolysis and anemia. The presence of dysfunctional erythroid progenitors causes ineffective erythropoiesis (Rivella, 2012) and impacts on hematopoietic stem cells (HSCs) self-renewal (Aprile et al., 2020).

The definitive cure for β-hemoglobinopathies is HSC transplantation, a treatment available only for the few patients who have an HLA-matched donor. Despite intense efforts, the only drug of some efficacy remains hydroxyurea (Yu et al., 2020), used to treat SCD (Platt, 2008) and, less successfully, β-thalassemia (Koren et al., 2008; Pourfarzad et al., 2013). Although the condition of β-diseased patients have greatly improved in the last years (Taher et al., 2009), there is a clear need of new approaches, the most innovative of them being based on genome modifications.

## The Lesson From Nature: Hereditary Persistence of Fetal Hemoglobin

The term hereditary persistence of fetal hemoglobin (HPFH) indicates a heterogeneous spectrum of spontaneous mutations, collectively named by their effect, i.e., the maintenance of the expression of fetal γ-globin in adult stages (Forget, 1998). HPFH alleles, when coinherited with β-hemoglobinopathies, greatly improve the condition of patients, 30% of HbF expression being considered a significant curative threshold able to prevent α free chains polymerization in β-thalassemias and HbS precipitation in SCD (Steinberg et al., 2014). Moreover, δβ-thalassemias, in which δ and β genes are deleted and γ-globin is reactivated, in general to a lesser extent than in HPFH, show that even a relatively low level of γ-globin has beneficial effects on β-thalassemias (Ottolenghi et al., 1982).

HPFH mutations can be broadly divided in three categories: large deletions affecting the structure of the β-locus; point mutations within the γ promoter that identify “hot-spot” HPFH sequences (−200, −175, −158, -distal CCAAT box); and mutations non-linked with the β-locus (Forget, 1998). Two of these non-linked loci, identified by genome-wide association analysis (GWAS), correspond to BCL11A gene, the most important repressor of γ-globin (Menzel et al., 2007; Sankaran et al., 2008; Uda et al., 2008) and to its key activator KLF1 (Borg et al., 2010; Zhou et al., 2010). More recently, knock-out studies in HUDEP cells led to the identification of LRF gene (also known as ZBTB7A), which represses γ-globin independently from BCL11A (Masuda et al., 2016). HPFH mutations within the β-locus greatly increase the expression of one or both γ genes in cis, whereas non-linked HPFH are associated with lower γ-globin levels (Forget, 1998).

The integration of the genetic data on HPFH with molecular studies led to the identification of the target sequences amenable for therapeutic genome editing (see below). In 1992, a pioneer study in mice transgenic for the human β-locus first demonstrated that it is indeed possible to reproduce HPFH (Berry et al., 1992).

## Therapeutic Genome Modifications: The Choice of the Modification

**Figure 1:**
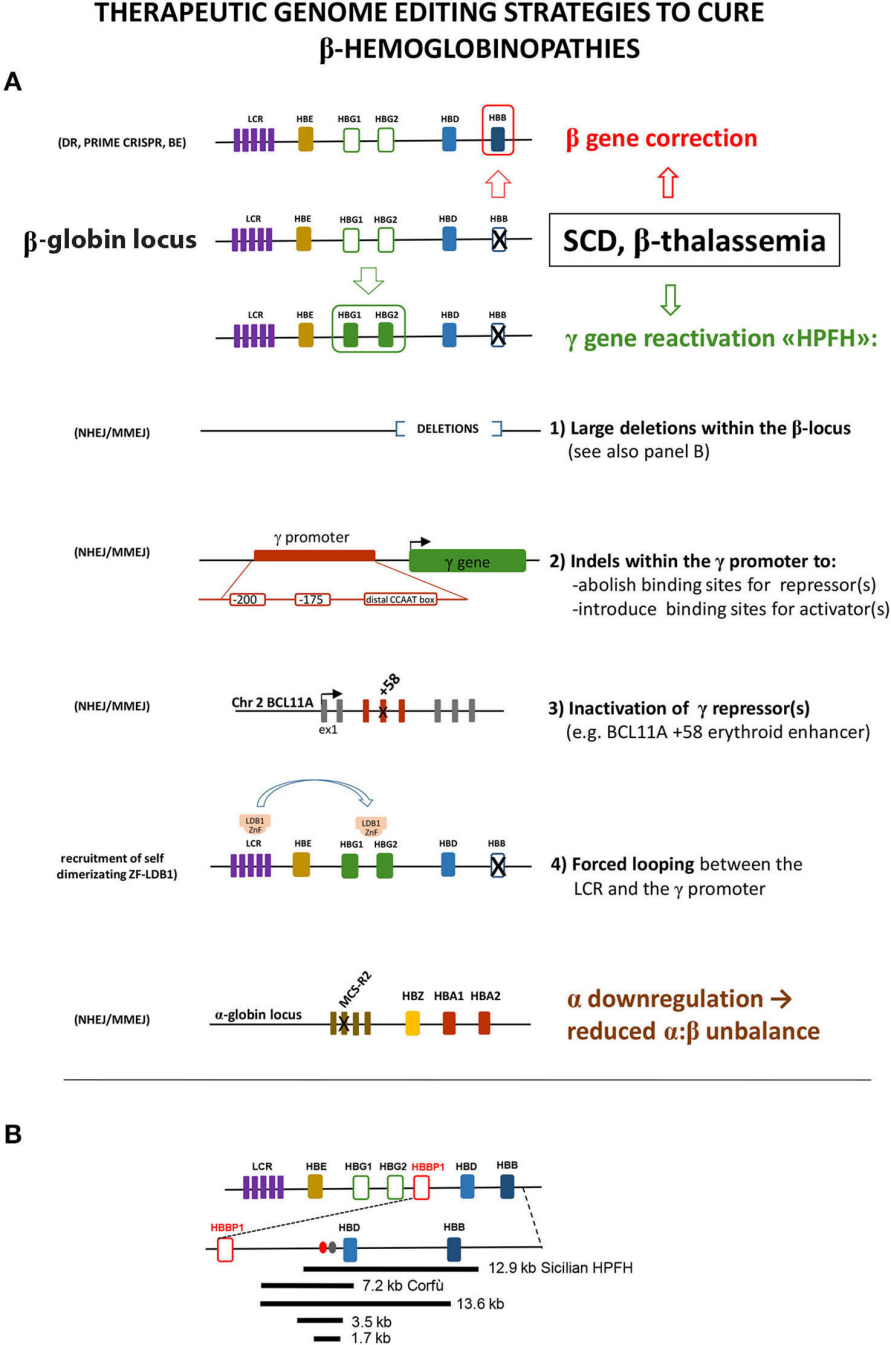
(**A**) Schematic representation of the different editing strategies developed to cure β-hemoglobinopathies. These approaches are oriented to correct the mutated β-globin gene, to reactivate the expression of the fetal γ gene in adult cells or to lower α-globin expression. The reactivation of γ can be obtained via the introduction of different modifications mimicking HPFH caused by large deletions within the β locus or by mutations within the γ promoter that disrupt the binding site of a repressor or create the binding site of an activator. As an alternative, the disruption of the erythroid-specific enhancer of the γ repressor BCL11A abolishes its expression in erythroid cells, thus resulting in γ overexpression. A different approach relies on the forced interaction of the LCR with the γ promoter obtained by exploiting the self-dimerization property of a fusion ZnF-LDB1 protein recruited on these regions. The detrimental effect of the α:β chain imbalance can be mitigated by mutating the MCS-R2 α-globin enhancer, in order to reduce α expression. These different strategies exploit different enzymes/cellular pathway described in the text: HDR, homology-directed repair; BE, base editing; NHEJ, non-homologous end joining; MMEJ, microhomology end joining. **(B)** Schematic representation of the deletions within the β-locus discussed in the text. Ovals represent the BCL11A (red) and the polypyrimidine (gray) sites thought to be responsible for γ repression.

In principle, different therapeutic genomic modifications can be envisaged to cure β-hemoglobinopathies (Figure 1A):

(1) The addition to the defective cell of an intact β gene, that, once delivered and integrated in the genome of the target hematopoietic stem cell, will produce the missing β chain under the control of an exogenous regulatory cassette, designed to ensure stable, erythroid-specific, and high-level expression. This approach (not discussed in this review), thanks to intensive efforts in developing safe and efficient vectors and in the improvement of their delivery, reached very significant results (Ikawa et al., 2019; Magrin et al., 2019). Importantly, the optimization of protocols developed by gene addition approaches represents a knowledge asset fundamental for bringing genome editing approaches to clinical application.

(2) The exact correction of the mutation causing the disease (gene editing), with the advantage of having the β gene expressed under the endogenous regulatory sequences, thus ensuring a perfectly regulated and stable expression with no risk of insertional mutagenesis. This procedure is particularly attractive for point mutations such as the β6Glu>Val SCD-causing mutation or for some β-thalassemia mutations, with the caveat that their extreme heterogeneity would require the design of patient-specific editing strategies. Under this aspect, this approach is worth only for mutations with high frequencies in given populations, as for the *HBB* −28A>G β-thal mutation in Southeast Asia or for the β^0^39C>T and β^+^thal IVS-I-110G>A mutations in the Mediterranean area (https://www.ithanet.eu/) (Kountouris et al., 2014).

In an extended perspective, the substitution of the mutated β gene with a wild-type β gene by homologous recombination could combine gene addition and gene editing to create a “universal” substitution cassette, which would minimize the risk of insertional mutagenesis (Cai et al., 2018).

(3) The introduction within the genome of modifications mimicking HPFH, in order to reactivate the expression of the fetal γ-globin gene and to compensate for the missing/defective β-globin expression.

(4) The reduction of the expression of α-globin, an important β-thalassemia modifier, as demonstrated by the milder clinical outcome of patients coinheriting α- and β-thalassemia (Thein, 2008; Mettananda et al., 2015). Reduced α levels indeed reduce the α:β chain imbalance, which represent a major problem in β-hemoglobinopathies. This effect has been achieved experimentally by deleting the MCS-R2 α enhancer (Mettananda et al., 2017).

## The Advent of Programmable Endonucleases in the Editing of Globin Genes: the Search for the Best Compromise Between Precision and Efficiency

In 1985, Oliver Smithies first exploited homologous recombination (HR) to introduce an exogenous DNA sequence within the β-locus (Smithies et al., 1985), demonstrating the feasibility of this approach. Since then, HR was used to generate gene knock-out models (including the KO of GATA1 (Pevny et al., 1991) and KLF1 (Nuez et al., 1995; Perkins et al., 1995)), by inserting exogenous DNA in the desired target. However, the very low efficiency of gene targeting, the consequent need of selecting the modified cells, and the technical difficulties of the method discouraged clinical applications (Vega, 1991).

The scenario radically changed with the advent of programmable endonucleases: zinc finger (ZnF) and TALENs first and now, CRISPR/Cas9 and its derivatives (Cornu et al., 2017; Komor et al., 2017). These nucleases introduce double-strand breaks (DSBs) with extreme specificity at the target genomic position. CRISPR/Cas9 is the most flexible system: its cutting specificity relies on a short guide RNA (sgRNA) and only requires the additional presence of an adjacent genomic proto-spacer adjacent motif (PAM) for its cut [this limit is actually being solved by the “near-PAM-less"-engineered CRISPR-Cas9 variants (Walton et al., 2020)]. In order to minimize possible off targets, different solutions are under study: better algorithms for the prediction of optimal DNA targets, optimized sgRNAs, engineered proto-spacers and Cas enzymes improved on the basis of thermodynamical models (Chen, 2019).

Once generated, DSBs are resolved by different DNA repair cellular pathways:

(i) The homology-directed repair (HDR) high-fidelity system that uses a donor template (the sister identical chromatid in physiological conditions) to repair DSBs when cells are in S and G2 phases. The implication is that HDR is poorly efficient in non-dividing HSC (Dever and Porteus, 2017), the target cell for therapeutic correction of β-hemoglobinopathies.

(ii) The non-homologous end joining (NHEJ) error-prone system acting in all cell cycle phases that inserts small indels at the site of the lesion, resulting in the disruption of the target sequence.

(iii) The microhomology end-joining (MMEJ) (Wang and Xu, 2017) error-prone system, which exploits small homology domains to align the broken filaments and close the gap. This molecular mechanism introduces deletions encompassing the microhomology regions flanking the break sites.

On these premises, the design of HDR recombination-based therapeutic strategies is difficult because of the requirement for a codelivered donor DNA template, of the low efficiency of HDR in HSCs and of the competition of the unwanted NHEJ and MMEJ error-prone repair systems. Despite these problems, the correction of the SCD mutation in HSCs was obtained by using both ZnF (Hoban et al., 2015) and CRISPR nucleases (Dever et al., 2016). However, the efficiency of the correction, assessed in HSCs *in vitro*, dramatically decreased after transplantation *in vivo*, confirming that HSCs are more resistant to HDR-based editing than more mature progenitors (Hoban et al., 2015) and that a selection step could be required to enrich for HSC-edited cells, capable of long-term correction *in vivo* (Dever et al., 2016). Instead, NHEJ is more flexible and allow to reach an efficiency up to ≈90% of edited HSCs that is maintained *in vivo* (Genovese et al., 2014; Chang et al., 2017; Charlesworth et al., 2018; Psatha et al., 2018; Wu et al., 2019).

The “perfect” editing should leave no trace, to avoid unintended off-target mutations and should at the same time guarantee high editing efficiency with reduced toxicity for HSCs. To reach this goal, an intense optimization work has been focused on the different steps of the genome editing procedure: the development of new editing reagents [single-strand DNA donor templates (Park et al., 2019), modified sgRNA (De Ravin et al., 2017; Park et al., 2019), pre-complexed ribonucleoproteins (RNPs) (Gundry et al., 2016)] and their integration in improved platforms for their delivery (Lino et al., 2018; Lattanzi et al., 2019; Schiroli et al., 2019). This massive effort finally led to the generation of selection-free HSCs of therapeutic potential (Genovese et al., 2014; DeWitt et al., 2016; Porteus, 2016; Yu et al., 2016; Wu et al., 2019).

## The Editing of Globin Genes Inspired by HPFH

NHEJ has been used to generate two classes of HPFH-inspired mutation: large deletions within the β-locus, to remove putative γ-globin repressive regions, and small indels within the γ-globin promoter or within the regulatory regions driving the erythroid expression of the γ-globin repressor BCL11A (Bauer et al., 2013; Canver et al., 2015). Large deletions focus around the critical “HPFH γδ-region” 5′ to the δ gene, deleted with different breakpoints in several HPFH [https://www.omim.org/entry/141749; https://www.ithanet.eu/; (Kountouris et al., 2014)]. This region is generally lost in deletions involving δ and β genes and causing HPFH, whereas it is retained in δβ-thalassemia deletions, which similarly remove δ and β genes but with little increase of γ-globin expression. This observation led to hypothesize that this region contains an element capable to repress the γ genes in *cis*. The CRISPR-mediated deletion corresponding to the 12.9-kb Sicilian HPFH, spanning from 3.2 kb upstream of the δ gene to the 3′ flanking region of the β gene, gave indeed a HPFH phenotype (increase in γ-globin with concomitant drop of β-globin expression) in HUDEP cells and in human *ex vivo* HSC-derived erythroblasts (Ye et al., 2016). The same result (γ-globin increased and β-globin decreased) was obtained by the CRISPR-mediated deletion (or inversion) of a large 13.6-kb region starting downstream to the pseudo-β1 (HBBP1) gene and extending into the β gene (Antoniani et al., 2018). The 5′ border of this deletion corresponds to the 5′ breakpoint of the Corfù δβ-thal 7.2-kb deletion (Wainscoat et al., 1985) that ends in the δ gene and is not associated with HPFH *in vivo* in humans (except in some rare cases, in homozygotes, in which an additional independent mutation in the downstream β gene is present (Kulozik et al., 1988). The CRISPR-mediated deletion of the 7.2-kb Corfù region and of two smaller internal regions of 3.5 and 1.7 kb, centered around a BCL11A binding site and a polypyrimidine stretch (Figure 1B), thought to mediate γ-globin repression (Sankaran et al., 2011), resulted in a very little γ-globin increase (Antoniani et al., 2018; Chung et al., 2019). These results indicate that the 1.7-kb element and its surrounding sequences *per se* are not an autonomous γ-globin silencer, as also suggested by previous studies (Galanello et al., 1990; Calzolari et al., 1999; Gaensler et al., 2003; Chakalova et al., 2005). Instead, they suggest a more complex scenario, where the competition with β-globin expression, the perfect distance/order between intergenic enhancer/repressor, and the enhancers delimitating the locus (the LCR and the 3′DNAseI hypersensitive site), all together concur to the correct γ/β gene expression (and to γ-globin increase, when perturbed in HPFH).

The effects of distorting the architecture of the β-locus can be turned in an advantage: Dr. Blobel and colleagues obtained a great increase in γ-globin (with β-globin reduction) by tethering LDB1 to the LCR and to the γ-globin promoter, thus forcing their looping (Deng et al., 2014). This result again highlights the importance of the competition between γ and β genes for the LCR.

HPFH mutation mapping within the γ-globin promoter alters the binding of transcription factors/cofactors. Theoretically, the γ-globin upregulation can be obtained either by increasing the binding of an activator or by decreasing the binding of a repressor. Both cases are observed in HPFH. Mutations at positions −198, −175, and −113 create new binding sites for erythroid transcriptional activators [KLF1 (Wienert et al., 2017), TAL1 (Wienert et al., 2015), GATA1 (Martyn et al., 2019), respectively]. Other mutations clustered around position −200 and around the distal CCAAT box (−115) reduce the binding of the γ-globin repressors LRF and BCL11A, respectively (Liu et al., 2018; Martyn et al., 2018).

Consistently, the CRISPR-mediated disruption of these two binding sites resulted in a relevant increase in γ-globin expression (Traxler et al., 2016; Weber et al., 2020). Of note, the editing of the −158 (“XmnI-Gγ-site”), known to be influenced by a QTL on chromosome 8 (Garner et al., 2002), only marginally increased γ-globin expression (Weber et al., 2020), suggesting that possible background effects might be taken into account when considering editing for therapeutic purposes.

The existence of two highly homologous γ-globin genes poses specific editing issues: the double-stranded DNA cut at the gRNA recognition sites in the HBG2 and HBG1 promoters could result in NHEJ-mediated joining of the two ends with loss of the intergenic (≈5 kb) genomic sequence in variable proportion (Traxler et al., 2016; Antoniani et al., 2018). Thus, the editing of these γ-globin regions can result either in the mutation of a single or both HBG genes or in the deletion of the intergenic region, with different resulting percentages of γ-globin induction. Moreover, given the presence of short repeats within the promoter, MMEJ can also occur (Traxler et al., 2016; Weber et al., 2020).

NHEJ can also be used to destroy the specific erythroid expression of repressors, such as BCL11A or, in principle, of LRF (both proteins have important roles in other hematopoietic cell types that must be preserved). On this front, four clinical trials based on targeting a GATA1-binding site within the intronic +58 (Canver et al., 2015) erythroid-specific BCL11A enhancer are ongoing (Hirakawa et al., 2020).

Theoretically, all the different genes involved in the γ-globin repression identified so far, including BCL11A, LRF, SOX6, and DRED are possible targets for genome editing, with the general caveat that their ablation should not perturb stem cell viability, their engraftment and differentiation potential. For example, the ubiquitous knockdown of BCL11A impairs normal HSC function and lymphopoiesis (Luc et al., 2016); LRF (Maeda et al., 2009), SOX6 (Cantu et al., 2011), and KLF1 (Nuez et al., 1995; Perkins et al., 1995) are instead required for proper erythroid differentiation. In this latter case, the need of fine-tuning the downregulation of the γ-globin repressor in order to lead to an appreciable γ-globin increase while maintaining a correct erythroid differentiation could represent an insurmountable obstacle.

Overall, the success obtained in reactivating γ-globin expression at therapeutic levels demonstrates that this strategy can work. Theoretically, the possibility to generate multiple HPFH mutations could further increase γ-globin expression.

Importantly, beside the final goal of its clinical application, the relative ease-of-use of the CRISPR-based editing techniques represents a formidable tool to answer the unsolved questions on the molecular mechanisms regulating the hemoglobin switching.

## Beyond Crispr/Cas9: Prime-CAS and BE-Cas

The use of HDR to correct β-disease mutations is limited by its low efficiency and by the downstream activation of p53, which can induce toxicity and, even worse, the possible selection of potentially harmful p53^low^ cells (Haapaniemi et al., 2018; Ihry et al., 2018). To overcome this problem, a new generation of engineered Cas9 that do not introduce DBSs (also avoiding unwanted NHEJ/MMEJ events triggered by the DBSs) and do not require donor DNA are under development. They rely on catalytically inactive Cas9 fused to a modified reverse transcriptase (prime editing) or to base-specific DNA deaminase enzymes [base editors (BEs)]. As for many innovations, these newcomers in the CRISPR toolbox have been tested on β-disease mutations. Prime editing has been used to correct the β6Glu>Val SCD mutation artificially introduced in HEK-293T cells (Anzalone et al., 2019). Dr. Bauer and colleagues recently demonstrated the versatility of BE by disrupting the GATA1-binding site within the +58 BCL11A erythroid-specific enhancer. The obtained HSC-edited cells express HbF at levels similar to those obtained by the NHEJ-mediated disruption of the same site and are capable of multi-lineage repopulation in serial transplantation experiments (Zeng et al., 2020). In addition, the simultaneous multiplex edit of the β-thal −28 A>G mutation in the TATA box of the β promoter increased β-globin production in the same cells.

Instead, Beam Therapeutics recently presented data relative to two therapeutic approaches based on BE, the first recreating an HPFH mutation and the second converting HbS into HbG-Makassar, a naturally occurring human variant that does not cause sickling^1^.

Although at present the issues of unwanted bystander/off target mutations remain to be explored, it is clear that Prime editing and BE represent important new instruments for genome editing, with the perspective to become even more attractive with the ongoing development of BE enabling more transition substitutions (Komor et al., 2018).

## Concluding Remarks

**Figure 2:**
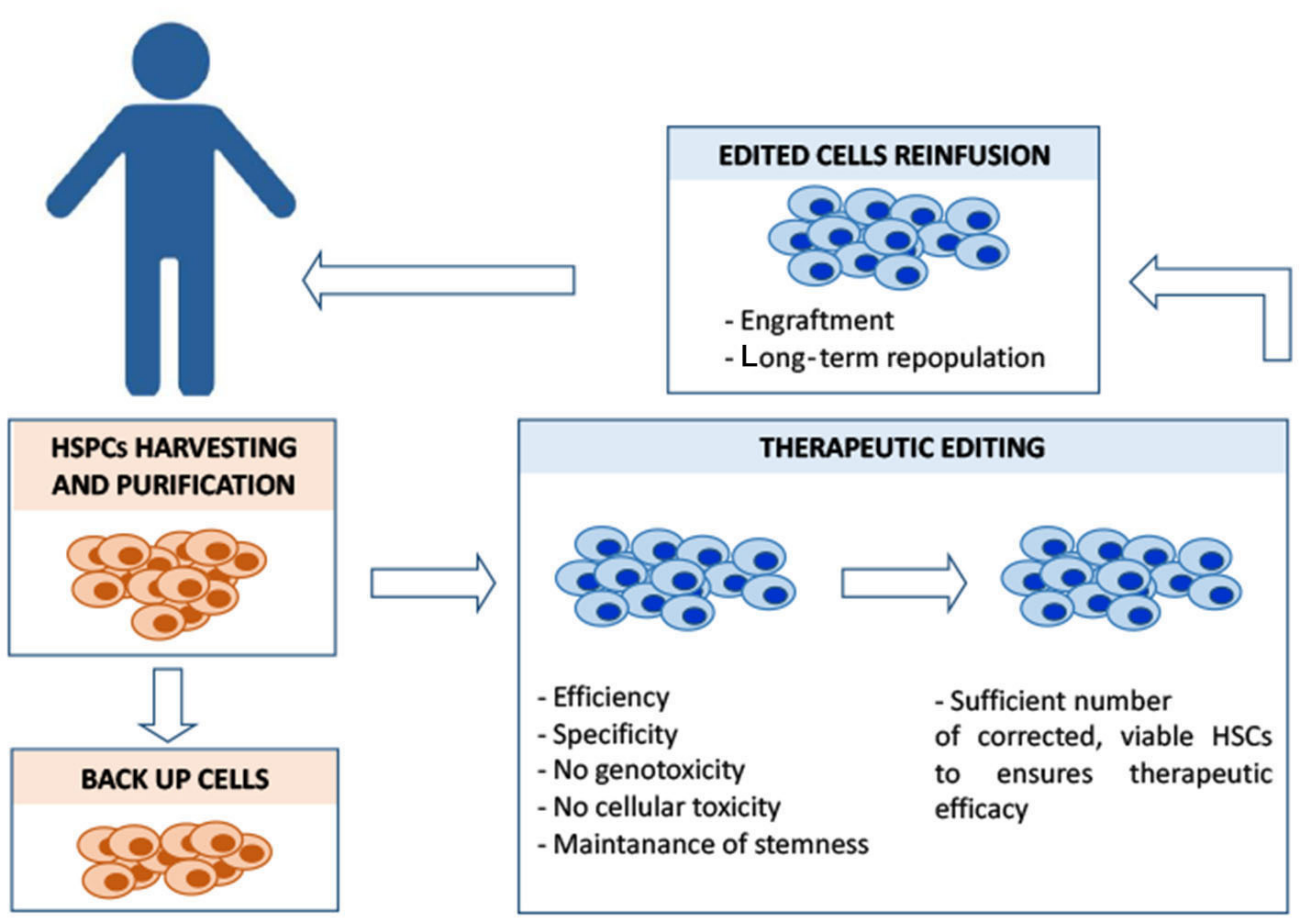
Overview of the different steps of HSC autologous transplantation and of its major critical issues. In the case of β-hemoglobinopathies, ineffective erythropoiesis and a compromised bone marrow microenvironment sensibly reduce the yield of CD34^+^HSCs amenable for the editing process, posing a serious problem in a clinical-scale setting. Different mobilization protocols are currently used to maximize the yield of harvested hematopoietic stem progenitor cells (HSPCs) that must also include backup cells to be reinfused into the patient in case of engraftment failure. Editing should ensure efficiency (in terms of complete allelic correction and percentage of edited cells) and, at the same time, minimize the exposure to editing reagents, to reduce the risk of unwanted mutations. Editing manipulations must preserve the population of CD34^+^ long-term repopulating cells. Before the reinfusion of the edited cells, the patient is treated with myeloablative agents to maximize the engraftment of the edited cells within the bone marrow niche. The conditioning regimen should be designed to guarantee the optimal risk-benefit balance between toxicity and efficacy of the engraftment, in order to achieve a stable, long-term therapeutic bone marrow repopulation.

The number of genome-editing tools is rapidly increasing (Papasavva et al., 2019; Doudna, 2020), holding the promise to reach in the near future a safe, precise, and efficient editing of β-disease mutations, via different strategies. The availability of different molecular options (HDR, NHEJ, and BE based, Figure 1) poses the problem of the evaluation of the pros and cons of each strategy (Ikawa et al., 2019; Papasavva et al., 2019): HDR-based approaches could ensure a higher precision at the expenses of HSC correction efficiency, whereas NHEJ is more efficient but less precise. Base editing, which does not require double-strand breaks, could be a safer option when a nucleotide substitution is required. Beside the choice of the optimal genetic modification, other issues remain open, first of all those related to unforeseeable genotoxicity (with the serious concern of inducing hyperproliferative/leukemic mutations in HSCs), the efficiency of the correction and the optimization of the delivery of genome editing reagents to target cells in conditions that preserve their stemness. Moreover, the clinical translation of these approaches requires the definition of scalable protocols to obtain under non-invasive conditions, a sufficient number of autologous HSCs amenable for the editing procedures and capable of optimal engraftment (in addition to backup cells to be reinfused in the patient in the case of engraftment failure) (Figure 2). This last point involves the identification of the best preparative conditioning regimen of the patient to allow efficient engraft of the corrected HSCs within the recipient niche (Psatha et al., 2016). Despite these difficulties, the recent announcement of the curative response of the first three patients (carrying a transfusion-dependent β-thalassemia and the SCD mutation) with CRISPR-Cas9-edited cells targeting BCL11A (CRISPR Therapeutics and Vertex CTX001 clinical trial^2^^&^^3^), clearly highlights the clinical potential of gene therapy. The advent of this new era urges the need to make these approaches affordable and available in low-resource settings/countries, where a large number of patients is waiting for a cure.

## Author Contributions

AR conceived and wrote the manuscript. GB contributed with ideas and discussion. GB and AŁ contributed in the organization of the manuscript. All authors contributed to the article and approved the submitted version.

## Conflict of Interest

The authors declare that the research was conducted in the absence of any commercial or financial relationships that could be construed as a potential conflict of interest.

## Footnotes

^1^ https://investors.beamtx.com/news-releases/news-release-details/beam-therapeutics-reports-additional-data-asgct-annual-meeting

^2^https://www.globenewswire.com/news-release/2020/06/12/2047260/0/en/CRISPR-Therapeutics-and-Vertex-Announce-New-Clinical-Data-for-Investigational-Gene-Editing-Therapy-CTX001-in-Severe-Hemoglobinopathies-at-the-25th-Annual-European-Hematology-Associ.html (accessed October 5, 2020).

^3^http://ir.crisprtx.com/news-releases/news-release-details/crispr-therapeutics-and-vertex-pharmaceuticals-announce-priority (accessed October 5, 2020).
